# Best-Evidence Systematic Review and Meta-Analysis of Endoscopic Carpal Tunnel Release Outcomes

**DOI:** 10.1016/j.jhsg.2023.07.011

**Published:** 2023-08-29

**Authors:** Larry E. Miller, Warren C. Hammert, Kevin C. Chung

**Affiliations:** ∗ Miller Scientific, Johnson City, TN; † Department of Orthopaedic Surgery, Division of Hand Surgery, Duke University Medical Center, Durham, NC; ‡ University of Michigan Comprehensive Hand Center, Michigan Medicine, Ann Arbor, MI

**Keywords:** Best evidence, Carpal tunnel release, Carpal tunnel syndrome, ECTR, Endoscopic

## Abstract

**Purpose:**

The aim of this systematic review and meta-analysis was to evaluate the safety and effectiveness of endoscopic carpal tunnel release (ECTR) using best-evidence synthesis methods.

**Methods:**

A systematic search of multiple databases was conducted for prospective contemporary studies published between January 2013 and January 2023 with at least 50 ECTR cases. Outcomes included the Quick Disabilities of the Arm, Shoulder, and Hand Questionnaire (Q-DASH) measured on a 0–100 scale, Boston Carpal Tunnel Questionnaire Symptom Severity Scale (BCTQ-SSS) and Functional Status Scale (BCTQ-FSS) on a 1–5 scale, pain visual analog scale on a 0–10 scale, conversion to open carpal tunnel release (CTR), complications, and reoperations. Outcomes were analyzed using a random-effects meta-analysis model. Metaregression was used to determine the association of patient- and study-level factors with ECTR outcomes.

**Results:**

A total of 17 studies with 1,632 patients treated with ECTR were included. Median follow-up durations ranged from 4 to 7 months depending on the outcome. Statistically significant and clinically important improvements were noted after ECTR for Q-DASH, BCTQ-SSS, BCTQ-FSS, and pain visual analog scale scores, with mean differences from baseline of −28.8, −1.8, −1.5, and −5.1, respectively (*P* < .001 for all). In metaregression, the strongest predictor of improvement in Q-DASH, BCTQ-SSS, and BCTQ-FSS was a greater preoperative score for that variable (all *P* ≤ .005), indicating that patients with worse symptoms improved the most. The risks of conversion to open CTR, complications, and revision CTR were 0.7%, 0.7%, and 0.5%, respectively.

**Conclusions:**

In a best-evidence synthesis of contemporary studies, ECTR resulted in significant improvements in function and pain, with a low risk of conversion to open surgery, complications, and reoperations over short-term follow-up.

**Clinical relevance:**

Patients treated with ECTR can expect generally favorable clinical outcomes over the short term. However, long-term outcomes after ECTR are not well characterized.

Carpal tunnel syndrome (CTS) is a common peripheral compressive neuropathy that affects millions globally.^1^, ^2^, ^3^, ^4^ Carpal tunnel release (CTR) is a definitive treatment option for patients with symptoms that are unresponsive to conservative treatment, resulting in clinical improvement in more than 90% of individuals.^3^, ^4^, ^5^ Currently, different CTR techniques are used in clinical practice including open CTR (OCTR), mini-open CTR, CTR with ultrasound guidance, and endoscopic CTR (ECTR), each with benefits and drawbacks.^6^^,^^7^

Endoscopic CTR makes one or two short incisions to introduce instruments for visualizing and dividing the transverse carpal ligament. The single-portal ECTR technique was first developed by Okutsu et al,^8^ followed by Chow’s^9^ dual-portal technique. Although clinical outcomes of OCTR and ECTR are generally comparable, patients tend to prefer ECTR^10^ for decreased postoperative pain, faster postoperative recovery, and reduced scar sensitivity. However, ECTR is associated with a distinct learning curve for surgeons^11^^,^^12^ and higher rates of transient nerve injury compared with OCTR.^13^^,^^14^

Numerous meta-analyses of randomized controlled trials have compared the safety and effectiveness of ECTR and OCTR.^13^, ^14^, ^15^, ^16^, ^17^, ^18^, ^19^, ^20^ The results of such meta-analyses highlight group comparisons but often provide limited insights into expected results within individual treatment groups. Supplementing the findings of comparative meta-analyses of randomized trials with results from individual treatment groups can potentially offer patients and physicians clearer and more practical information. This is particularly important because a substantial portion of peer-reviewed literature is based on real-world evidence.^21^ Therefore, the purpose of this systematic review and meta-analysis was to summarize the safety and effectiveness outcomes from contemporary ECTR literature by adhering to best-evidence principles to mitigate potential biases and increase the reliability of the results.

## Materials and Methods

This contemporary best-evidence systematic review and meta-analysis was prospectively registered at www.researchregistry.com (reviewregistry1649). The methods and reporting adhered to the Preferred Reporting Items for Systematic Reviews and Meta-Analyses^22^ and the best-evidence synthesis methods proposed by Slavin,^23^ which ensures that the conclusions are drawn from the most methodologically rigorous studies available.

### Study eligibility criteria

The inclusion criteria for this best-evidence review required studies to be prospective, treat at least 50 hands with ECTR, report at least one outcome specified in this review within the 5-year postoperative follow-up period, and be published between January 2013 and January 2023, regardless of language. Studies were excluded if they reported outcomes from various CTR techniques, had ECTR performed with concomitant surgical procedures, had revision ECTR performed, or were published as abstracts or duplicate publications. The rationale for these best-evidence criteria is provided in Supplementary Table 1, available online on the Journal’s website at https://www.jhsgo.org.

### Search strategy and study selection process

A systematic search was conducted across multiple electronic databases, including MEDLINE, Embase, and the Cochrane Central Register of Controlled Trials. The search strategy was purposely broad to ensure maximum search sensitivity. We used the terms [ECTR or endoscop∗] and [carpal or CTS or median nerve] for the MEDLINE searches. The search strategies were adapted as necessary for other databases. We also conducted supplementary manual searches in the Directory of Open Access Journals and Google Scholar and in the reference lists of included articles and relevant review articles. Two researchers with expertise in systematic reviews independently screened articles for eligibility. All potentially relevant studies were obtained in full text and reviewed. Discrepancies in study eligibility were resolved through discussion and consensus. The final search was conducted in February 2023.

### Data extraction and outcomes

The researchers independently extracted data from eligible studies using standardized data collection forms. Data extraction discrepancies were resolved through discussion and consensus. We evaluated the methodological quality of studies using the National Institutes of Health assessment tool for before–after studies.^24^ The main outcomes of this meta-analysis were the Quick Disabilities of the Arm, Shoulder, and Hand Questionnaire (Q-DASH), Boston Carpal Tunnel Questionnaire Symptom Severity Scale (BCTQ-SSS) and Functional Status Scale (BCTQ-FSS) scores, pain visual analog scale (VAS), and the rates of conversion to OCTR, complications, and reoperations. The Q-DASH was analyzed on a 0 to 100 scale, the BCTQ scales on a 1 to 5 scale, and pain VAS on a 0 to 10 scale. Because these outcomes were reported at multiple time points in some studies, the values at the final follow-up interval in each study were included in the analysis. The minimal clinically important differences (MCIDs) for the postoperative change in patient-reported outcomes were −15 points for Q-DASH,^25^ −1.14 points for BCTQ-SSS,^26^ −0.74 points for BCTQ-FSS,^26^ and −2 points for pain VAS.^27^ Conversion to OCTR, complications, and reoperations were analyzed using all events reported during the postoperative follow-up period.

### Statistical methods

Continuous outcomes were analyzed using the mean difference from baseline and 95% confidence interval (CI) for individual studies and the overall estimate. Binary outcomes were reported using the event rate and 95% CI. The overall results were obtained using a restricted maximum likelihood random-effects meta-analysis to account for heterogeneity among studies. In studies that reported outcomes from multiple ECTR subgroups, we combined the data; hence, each study provided only one ECTR group for each outcome.^28^ Heterogeneity of outcomes among the studies was estimated with the *I*^2^ statistic where a value of 0% represented no heterogeneity, and larger values represented increasing heterogeneity.^29^ Potential publication bias was assessed by the visual examination of funnel plot symmetry and using the trim-and-fill method, which recalculated the meta-analysis results based on the estimated number of studies missing due to publication bias.^30^ A one-study-removed meta-analysis assessed the influence of single-study effects on outcomes. Finally, we used metaregression to evaluate the association of patient- and study-level factors with ECTR outcomes reported in at least five studies and with significant observed heterogeneity (*I*^2^ > 50%). The independent variables of interest were age, sex, the preoperative value for patient-reported outcomes, the number of surgical incisions, follow-up duration, and sample size.

## Results

### Patient and study characteristics

Of the initial 536 titles and abstracts identified in the searches, 333 were deemed irrelevant and subsequently excluded. Among the remaining studies, the most common reasons for exclusion were insufficient sample size (48), review article (41), retrospective study design (38), studies that did not use ECTR or reported outcomes from various CTR techniques (19), and database analyses (19). Ultimately, the meta-analysis included 17 studies,^31^, ^32^, ^33^, ^34^, ^35^, ^36^, ^37^, ^38^, ^39^, ^40^, ^41^, ^42^, ^43^, ^44^, ^45^, ^46^, ^47^ with 1,632 patients from 13 countries, representing the best evidence on ECTR published over the past decade (Supplementary Figure 1, available online on the Journal’s website at https://www.jhsgo.org). The median age of participants was 54 years (with a study-wide mean range of 43–65 years), with a higher proportion of female patients (median 70%; with a study-wide mean range of 34% to 95%), and a median CTS symptom duration of 24 months (with a study-wide mean range of 1–72 months; Table 1). The mean preoperative patient-reported outcome values were 43.1 (95% CI: 33.4–52.8) for Q-DASH, 3.1 (95% CI: 2.7–3.5) for BCTQ-SSS, 2.9 (95% CI: 2.4–3.5) for BCTQ-FSS, and 6.5 (95% CI: 5.5–7.5) for pain VAS. The methodological quality of all studies was rated as good (Supplementary Table 2, available online on the Journal’s website at https://www.jhsgo.org), validating the utility of the best-evidence study selection criteria.

**Table 1:** Study and Patient Characteristics

Study	Surgery Dates	Country	Patients	Hands	Age (y)	Female (%)	Symptom Duration (mo)	Surgical Incisions	Dominant Side Treated (%)	Simultaneous Bilateral (%)
Chalidis (2013)^31^	2006–2010	Greece	85	170	57	73	43	1	100	100
Chandra (2013)^32^	2001–2008	India	100	105	46	86	27	2	—	0
Ecker (2015)^33^	—	Australia	50	—	57	34	—	1	—	—
Gurpinar (2019)^34^	2016–2018	Turkey	54	54	51	67	24	2	—	0
Ilyas (2019)^35^	2017–2018	USA	61	61	60	51	—	1	—	0
Jorgsholm (2021)^36^	—	Sweden	94	94	43	47	6	1	87	0
Nazerani (2014)^37^	2007–2012	Iran	176	—	48	92	1	1	—	—
Nguyen (2022)^38^	2019–2020	Vietnam	77	77	51	86	24∗	2	—	0
Okamura (2014)^39^	2009–2012	Brazil	78	80	55	95	72	1	52	3
Rivlin (2018)^40^	—	USA	84	—	—	—	—	1	—	—
Sato (2021)^41^	—	Japan	200	225	65	71	—	1	—	13
Schroeder (2022)^42^	—	USA	105	105	58	68	—	2	72	0
Teh (2021)^43^	2016–2018	Malaysia	46	50	59	80	—	1	—	9
Trung (2019)^44^	2016–2017	Vietnam	118	150	52	83	23	1	68	27
Tulipan (2018)^45^	—	USA	154	154	57	58	—	1	—	0
van Rooij (2022)^46^	2015–2019	Netherlands	81	162	52	58	—	1	100	100
Zhang (2016)^47^	2008–2010	China	69	69	48	64	6	2	—	0

∗ Estimated value.

### ECTR effectiveness

**Figure 1:**
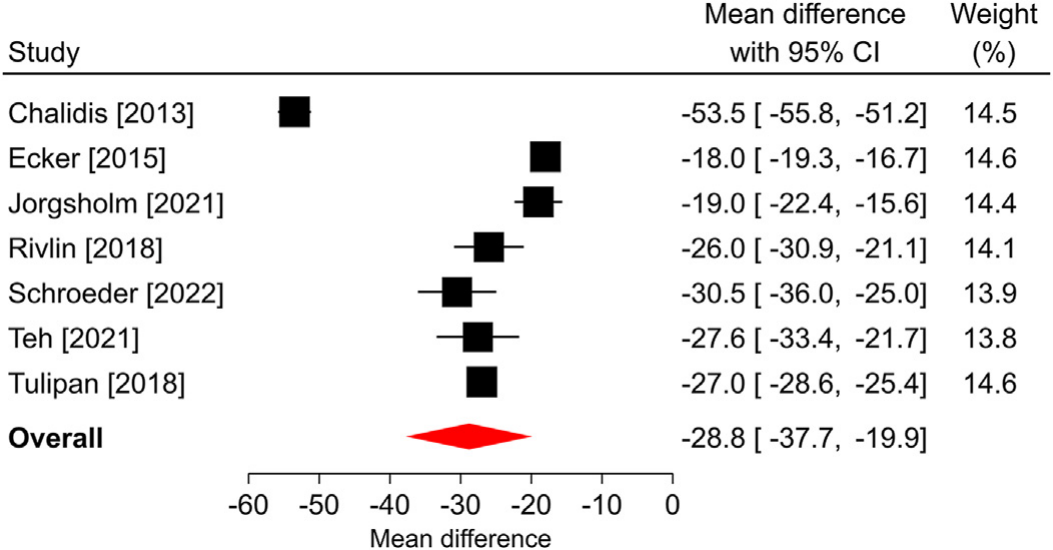
Forest plot of change in Q-DASH after ECTR. The mean difference from baseline and 95% CI are plotted for each study. The size of the square is proportional to the weighting of the study in the meta-analysis. The overall mean difference is denoted by the diamond apex, and the 95% CI is denoted by the diamond width. The overall mean difference was −28.8 (*P* < .001) over a median of 4-month follow-up. Significant heterogeneity (*I*^2^ = 99%) was identified among studies. Q-DASH, Quick Disabilities of the Arm, Shoulder, and Hand.

**Figure 2:**
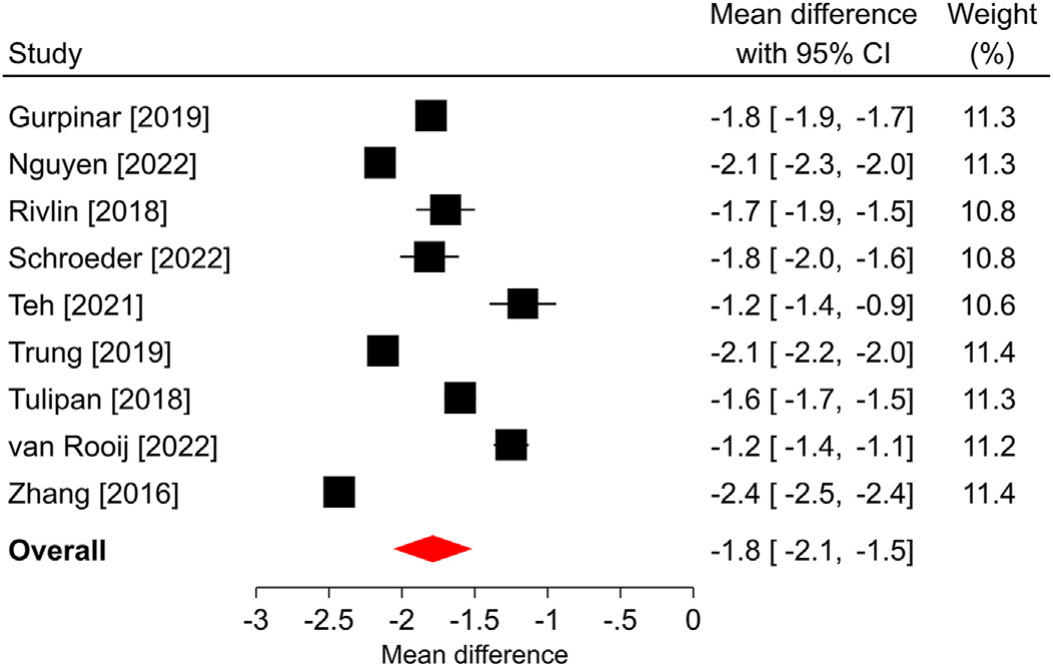
Forest plot of change in BCTQ-SSS after ECTR. The mean difference from baseline and 95% CI are plotted for each study. The size of the square is proportional to the weighting of the study in the meta-analysis. The overall mean difference is denoted by the diamond apex, and the 95% CI is denoted by the diamond width. The overall mean difference was −1.8 (*P* < .001) over a median of 6-month follow-up. Significant heterogeneity (*I*^2^ = 98%) was identified among studies. BCTQ-SSS, Boston Carpal Tunnel Questionnaire Symptom Severity Scale.

**Figure 3:**
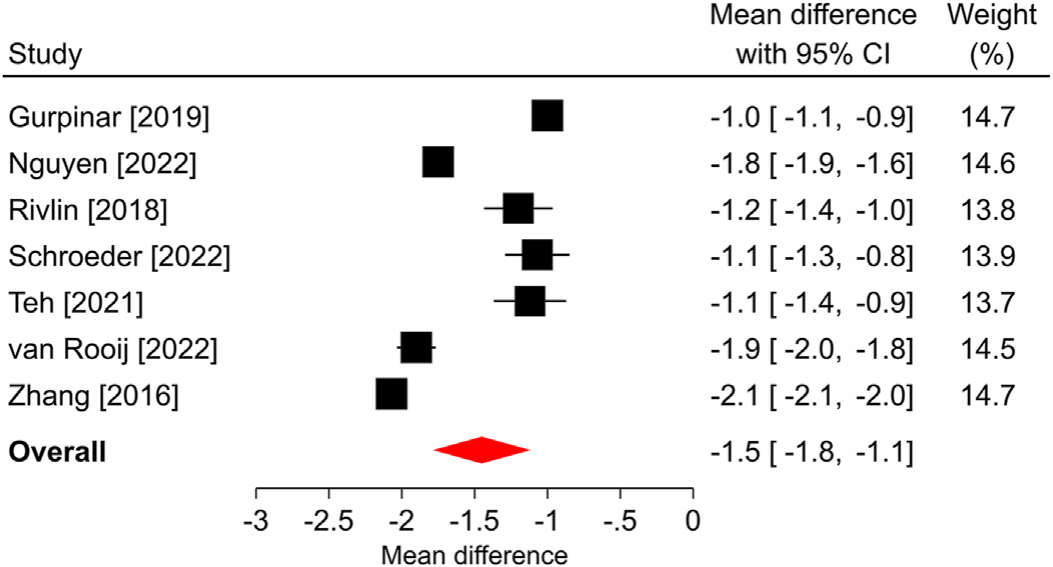
Forest plot of change in BCTQ-FSS after ECTR. The mean difference from baseline and 95% CI are plotted for each study. The size of the square is proportional to the weighting of the study in the meta-analysis. The overall mean difference is denoted by the diamond apex, and the 95% CI is denoted by the diamond width. The overall mean difference was −1.5 (*P* < .001) over a median of 6-month follow-up. Significant heterogeneity (*I*^2^ = 98%) was identified among studies. BCTQ-FSS, Boston Carpal Tunnel Questionnaire Functional Status Scale.

**Figure 4:**
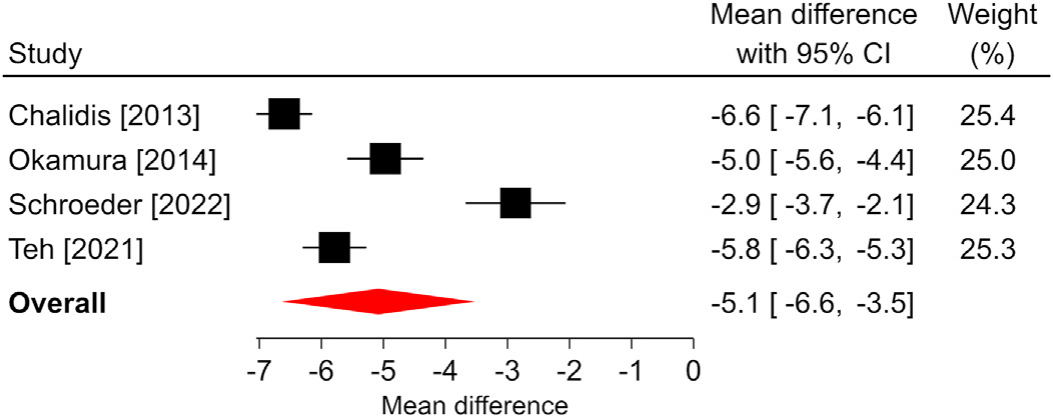
Forest plot of change in pain VAS after ECTR. The mean difference from baseline and 95% CI are plotted for each study. The size of the square is proportional to the weighting of the study in the meta-analysis. The overall mean difference is denoted by the diamond apex, and the 95% CI is denoted by the diamond width. The overall mean difference was −5.1 (*P* < .001) over a median of 6-month follow-up. Significant heterogeneity (*I*^2^ = 97%) was identified among studies. VAS, visual analog scale.

Among the seven studies reporting the change in Q-DASH scores over a median follow-up of 4 months, all reported statistically significant improvements compared with the preoperative scores. The overall mean difference was −28.8 points (95% CI: −37.7 to −19.9; *P* < .001), and significant heterogeneity was identified among the studies (*I*^2^ = 99%; Fig. 1). The BCTQ-SSS scores statistically improved in each of the nine studies reporting this outcome and in the overall analysis over a median of 6 months of follow-up. The overall mean difference was −1.8 points (95% CI: −2.1 to −1.5; *P* < .001), with significant heterogeneity identified among studies (*I*^2^ = 98%; Fig. 2). Similar findings were observed in the seven studies reporting BCTQ-FSS, where all reported statistically significant improvements over a median of 6 months of follow-up, the overall mean difference was −1.5 points (95% CI: −1.8 to −1.1; *P* < .001), and significant heterogeneity was observed (*I*^2^ = 98%; Fig. 3). Among the four studies reporting a change in pain VAS over a median of 6 months of follow-up, all reported statistically significant improvements compared with preoperative scores. The overall mean difference was −5.1 points (95% CI: −6.6 to −3.5; *P* < .001), with significant heterogeneity identified among studies (*I*^2^ = 97%; Fig. 4). The overall mean difference and the entire 95% CI exceeded the MCID for each of these outcomes, indicating that the improvements from baseline were not only statistically significant but also clinically important.

### ECTR complications

The short-term safety outcomes with ECTR were favorable. Conversion to OCTR was performed in 0.7% (95% CI: 0.3% to 1.2%) of the cases (Supplementary Figure 2, available online on the Journal’s website at https://www.jhsgo.org). Postsurgical complications were reported in 0.7% (95% CI: 0.2% to 1.2%) of the cases over a median follow-up duration of 7 months (Supplementary Figure 3, available online on the Journal’s website at https://www.jhsgo.org). The most common complication was pain/paresthesia (10 cases), all of which resolved within 6 months. Only one (0.1%) case of median nerve branch transection was reported,^37^ which was converted to OCTR. Reoperations were performed in 0.5% (95% CI: 0.1% to 0.8%) of the cases over a median follow-up duration of 6 months (Supplementary Figure 4, available online on the Journal’s website at https://www.jhsgo.org). Heterogeneity among studies was negligible for all complication outcomes (*I*^2^ ≤ 5%).

### Publication bias, sensitivity analysis, and metaregression

The trim-and-fill analysis resulted in identical estimates as the main analysis, except for reoperations that were 0.5% in the main analysis and 0.4% in the trim-and-fill analysis. Thus, publication bias was negligible for all outcomes, suggesting that the meta-analysis results were not influenced by the selective publication of studies based on the direction or strength of their findings. Additionally, no individual study substantially influenced the overall conclusions of the meta-analysis. By rerunning the meta-analysis excluding each study at a time, the overall mean differences ranged from −30.7 to −24.3 for Q-DASH (all *P* < .001), −1.9 to −1.7 for BCTQ-SSS (all *P* < .001), −1.5 to −1.3 for BCTQ-FSS (all *P* < .001), and −5.8 to −4.6 for pain VAS (all *P* < .001). As with the primary analysis, the overall mean difference and the entire 95% CI in the one-study-removed sensitivity analysis exceeded the MCID for each of these outcomes. Similarly, the overall event rates were comparable in the one-study-removed sensitivity analyses with values ranging from 0.6% and 0.9% for conversion to OCTR, 0.6% to 1.1% for complications, and 0.4% to 0.5% for reoperations. Three outcomes met the criteria for inclusion in the metaregression based on the number of studies and the presence of heterogeneity: Q-DASH, BCTQ-SSS, and BCTQ-FSS. For each of these outcomes, the baseline value was the strongest predictor of postoperative change, where more severe symptoms were associated with a greater magnitude of postoperative improvement (Table 2, Supplementary Figures 5–7, available online on the Journal’s website at https://www.jhsgo.org).

**Table 2:** Association of Patient- and Study-Factors on Outcomes Following ECTR∗

Variable	Q-DASH Mean Difference	BCTQ-SSS Mean Difference	BCTQ-FSS Mean Difference
Baseline score	*z* = −3.76 *P* < .001	*z* = −2.83 *P* = .005	*z* = −2.87 *P* = .004
Follow-up (mo)	*z* = −2.66 *P* = .008	*z* = −1.99 *P* = .05	*z* = −0.48 *P* = .63
No. incisions	*z* = −0.14 *P* = .89	*z* = −2.00 *P* = .05	*z* = −0.17 *P* = .87
Age (y)	*z* = −0.78 *P* = .44	*z* = 2.16 *P* = .03	*z* = 1.86 *P* = .06
Female (%)	*z* = −1.75 *P* = .08	*z* = −0.68 *P* = .50	*z* = 0.45 *P* = .65
Sample size	*z* = −2.80 *P* = .005	*z* = −1.59 *P* = .11	*z* = −2.45 *P* = .01

∗ Negative *z*-scores indicate that as the value for the baseline variable increases, the mean difference for the outcome decreases (ie, improves).

## Discussion

Several main findings were observed from this systematic review and meta-analysis of 17 contemporary prospective ECTR studies that employed best-evidence methodology to select the most methodologically rigorous studies. First, statistically significant and clinically important improvements were noted in postoperative function and symptoms after ECTR. Second, the magnitude of improvement in patient-reported outcomes was highly related to the preoperative value, indicating that patients with worse symptoms improved the most. Third, the rates of conversion to OCTR, complications, and reoperations were low (all <1%). Fourth, the outcomes reported in this meta-analysis were observed over relatively short follow-up durations, ranging from 4 to 7 months. Finally, most of the recent ECTR literature consists of studies of lower methodological quality as evidenced by the large number of studies excluded from this review due to retrospective designs and small sample sizes. Overall, although the short-term outcomes with ECTR appear favorable based on the best evidence in the literature, the overall quality of available ECTR research is suboptimal, underscoring the need for larger prospective studies to substantiate these findings over longer follow-up durations.

The findings of this systematic review and meta-analysis are consistent with those of the American Association of Orthopedic Surgeons that stated “limited evidence supports that if surgery is chosen, a practitioner might consider using endoscopic carpal tunnel release based on possible short-term benefits.”^48^ Indeed, limited high-quality evidence exists to support the long-term utility of ECTR. Perhaps, the best evidence comes from the study of Atroshi et al^49^ who reported similar symptom and function improvement, and reoperation rates with ECTR compared with OCTR after 13 years of follow-up. Additionally, Means et al^50^ reported that BCTQ-SSS and BCTQ-FSS scores remained stable from 6 months to 9 years after ECTR, suggesting that short-term outcomes after ECTR may reflect longer-term results. Still, notwithstanding these examples, most of the ECTR literature comprises short-term follow-ups, retrospective design, and/or small sample sizes, complicating data interpretation and providing limited information to patients contemplating their recovery after ECTR.

Despite the methodological limitations of the literature, ECTR utilization has been increasing, particularly in the Medicare population.^51^ However, the application of ECTR continues to vary widely based on geographical location and patient demographics. For example, ECTR comprises 6% of all CTR cases in Turkey,^52^ 25% to 26% in the United States,^53^^,^^54^ 30% in Japan,^55^ and 40% in France.^56^ Numerous factors potentially influence the decision to undergo ECTR and those affecting outcomes remain unclear. Although our analysis identified a strong association between preoperative symptoms and postoperative symptom improvement, other factors influencing patient outcomes could not be analyzed due to the limited number of included studies. Some studies have reported a learning curve with ECTR;^11^^,^^12^ however, these data were not reported in the studies we evaluated. Additionally, it is well known that preoperative patient expectations may vary tremendously among demographic groups,^57^ which could further influence outcome reporting in ECTR studies. Overall, this meta-analysis identified ECTR as a safe and effective treatment for CTS based on short-term results while providing greater symptom improvement in more severe cases. However, the factors contributing to preferences for ECTR and additional factors that may influence outcomes could not be adequately assessed in this review and warrant additional study.

Several limitations are associated with this review. First, although the results presented here were developed using best-evidence synthesis methods, they represent level IV evidence. Only two randomized trials compared ECTR with another CTR technique that met the best-evidence criteria,^34^^,^^47^ which was insufficient to perform a meta-analysis. Based on results from previous meta-analyses, the effectiveness of ECTR is expected to be comparable with other CTR techniques.^18^^,^^58^^,^^59^ The second relates to the limited follow-up duration in these studies. Although we extracted data at the final follow-up interval in each study, the median follow-up for the outcomes in this meta-analysis ranged from 4 to 7 months. Although most clinical improvements and complications after CTR typically occur during this period,^60^^,^^61^ additional longer-term data would be helpful to corroborate these early outcomes. Third, considerable variation exists in the types of outcomes reported in individual studies. Although complications and reoperations were reported in most studies, the additional outcomes reported in each study varied, and incompleteness of patient attrition reporting was a primary source of bias. Adherence to publishing standards such as the STROBE statement for observational studies^62^ or the CONSORT statement for randomized trials^63^ is highly recommended for future ECTR publications.

## Conclusions

In a best-evidence synthesis of contemporary studies, ECTR resulted in significant improvements in functional outcomes and pain relief, with a low risk of conversion to open surgery, complications, and reoperations over short-term follow-up.
