# Supplementary material for: Best-Evidence Systematic Review and Meta-Analysis of Endoscopic Carpal Tunnel Release Outcomes
Source: J Hand Surg Glob Online. 2023 Aug 29;5(6):768–73. doi: 10.1016/j.jhsg.2023.07.011 (PMC10721515; doi:10.1016/j.jhsg.2023.07.011)
Supplement: Supplementary Figures [file mmc1.docx]

**Supplement**

**Supplement Table 1**

Best-evidence Study Entry Criteria

| **Criterion** | **Rationale** |
| --- | --- |
| ***Inclusion*** |  |
| Prospective study | Minimizes risk of selection bias |
| Minimum 50 hands treated | Inclusion of larger studies reduces small-study effects such as selection bias, publication bias, and inflated effect sizes, thereby enhancing precision and generalizability of the meta-analysis findings |
| Reported at least one outcome | Necessary to develop meta-analysis estimates |
| No language restrictions | Reduces risk of language bias |
| Publication between 2013 and 2023 | Ensures that the meta-analysis incorporates the most current and relevant evidence |
| ***Exclusion*** |  |
| Various or unclear CTR technique | Ensures strict focus on outcomes with ECTR only |
| Concomitant surgical procedures | Minimizes confounding of results due to effects of the concomitant procedure |
| Revision ECTR study | Patient outcomes are inferior in revision vs. primary ECTR |
| Published as abstract only | Abstracts lack sufficient detail with which to evaluate methodological quality and determine the risk of bias accurately |
| Duplicate publication | Avoids duplication of data from common patients |

CTR, carpal tunnel release; ECTR, endoscopic carpal tunnel release.

**Supplement Table 2**

Methodological Study Quality Assessment using the National Institute of Health Assessment Tool for Before-After Studies ^*^

| **Study** | **1** | **2** | **3** | **4** | **5** | **6** | **7** | **8** | **9** | **10** | **11** | **12** | **Quality**  **Rating** |
| --- | --- | --- | --- | --- | --- | --- | --- | --- | --- | --- | --- | --- | --- |
| Chalidis [2013] ^31^ | Y | Y | Y | Y | Y | Y | Y | N | N | Y | N | ^†^ | Good |
| Chandra [2013] ^32^ | Y | Y | Y | Y | Y | Y | Y | N | Y | Y | N | ^†^ | Good |
| Ecker [2015] ^33^ | Y | Y | Y | Y | Y | Y | Y | N | N | Y | N | ^†^ | Good |
| Gurpinar [2019] ^34^ | Y | Y | Y | Y | Y | Y | Y | N | Y | Y | N | ^†^ | Good |
| Ilyas [2019] ^35^ | Y | Y | Y | Y | Y | Y | Y | N | Y | Y | N | ^†^ | Good |
| Jorgsholm [2021] ^36^ | Y | Y | Y | Y | Y | Y | Y | N | N | Y | N | ^†^ | Good |
| Nazerani [2014] ^37^ | Y | N | Y | Y | Y | Y | Y | N | Y | Y | N | ^†^ | Good |
| Nguyen [2022] ^38^ | Y | Y | Y | Y | Y | Y | Y | N | N | Y | N | ^†^ | Good |
| Okamura [2014] ^39^ | Y | Y | Y | Y | Y | Y | Y | N | N | Y | N | ^†^ | Good |
| Rivlin [2018] ^40^ | Y | Y | Y | Y | Y | Y | Y | N | N | Y | N | ^†^ | Good |
| Sato [2021] ^41^ | Y | Y | Y | Y | Y | Y | Y | N | Y | Y | N | ^†^ | Good |
| Schroeder [2022] ^42^ | Y | Y | Y | Y | Y | Y | Y | N | Y | Y | N | ^†^ | Good |
| Teh [2021] ^43^ | Y | N | Y | Y | Y | Y | Y | N | Y | Y | N | ^†^ | Good |
| Trung [2019] ^44^ | Y | Y | Y | Y | Y | Y | Y | N | N | Y | N | ^†^ | Good |
| Tulipan [2018] ^45^ | Y | Y | Y | Y | Y | Y | Y | N | Y | N | N | ^†^ | Good |
| van Rooij [2022] ^46^ | Y | Y | Y | Y | Y | Y | Y | N | Y | Y | N | ^†^ | Good |
| Zhang [2016] ^47^ | Y | Y | Y | Y | Y | Y | Y | N | N | Y | N | ^†^ | Good |

^*^Item numbers and associated descriptions include: 1) objective clearly stated; (2) eligibility criteria described; (3) representative patient population; (4) all eligible participants enrolled in study; (5) sufficient sample size; (6) intervention described; (7) outcome measures specified; (8) outcome assessors blinded; (9) loss to follow-up and intention-to-treat analysis; (10) statistical analysis of outcome measures before and after intervention; (11) interrupted time-series design; (12) individual data used for group-level effects.

^†^Not applicable.


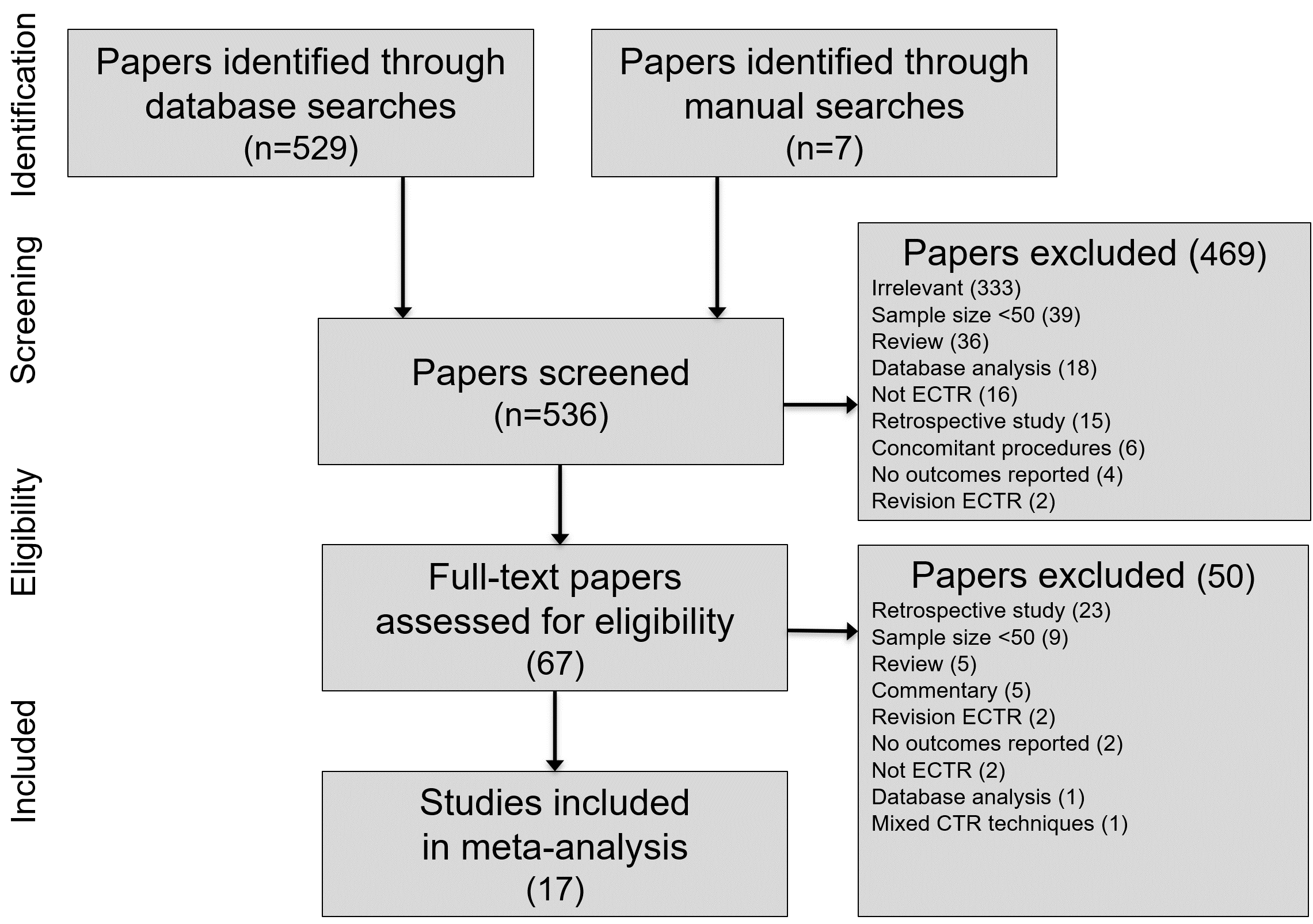


**Supplement Figure 1.** PRISMA flow diagram. CTR, carpal tunnel release; ECTR, endoscopic carpal tunnel release.

**Supplement Figure 2.** Forest plot of conversion from endoscopic to open carpal tunnel release. The proportion and 95% confidence interval are plotted for each study. The size of the square is proportional to the weighting of the study in the meta-analysis. The overall proportion is denoted by the diamond apex and the 95% confidence interval is denoted by the diamond width. The overall event rate was 0.7%. Negligible heterogeneity (*I*^2^=0%) was identified among studies. Note that in random effects meta-analysis of proportions, the estimated proportions for studies with no events may exceed zero due to shrinkage towards the pooled estimate, leveraging information from the overall estimate to enhance precision and stability.

**Supplement Figure 3.** Forest plot of complications after endoscopic carpal tunnel release. The proportion and 95% confidence interval are plotted for each study. The size of the square is proportional to the weighting of the study in the meta-analysis. The overall proportion is denoted by the diamond apex and the 95% confidence interval is denoted by the diamond width. The overall event rate was 0.7% over a median 7-month follow-up. Negligible heterogeneity (*I*^2^=5%) was identified among studies. Note that in random effects meta-analysis of proportions, the estimated proportions for studies with no events may exceed zero due to shrinkage towards the pooled estimate, leveraging information from the overall estimate to enhance precision and stability.

**Supplement Figure 4.** Forest plot of reoperations after endoscopic carpal tunnel release. The proportion and 95% confidence interval are plotted for each study. The size of the square is proportional to the weighting of the study in the meta-analysis. The overall proportion is denoted by the diamond apex and the 95% confidence interval is denoted by the diamond width. The overall event rate was 0.5% over a median 6-month follow-up. Negligible heterogeneity (*I*^2^=0%) was identified among studies. Note that in random effects meta-analysis of proportions, the estimated proportions for studies with no events may exceed zero due to shrinkage towards the pooled estimate, leveraging information from the overall estimate to enhance precision and stability.

**Supplement Figure 5.** Bubble plot of the association between the change in Q-DASH after endoscopic carpal tunnel release and baseline Q-DASH score. Black circles represent values of individual studies where the circle size is proportional to the study weight in the random-effects model. The red line represents the regression line of best fit with the shaded area indicating the 95% confidence interval. The regression equation to predict Q-DASH change = [21.6 – (1.2 * Baseline Q-DASH)]; p<0.001. Q-DASH, Quick Disabilities of the Arm, Shoulder, and Hand.

**Supplement Figure 6.** Bubble plot of the association between the change in BCTQ-SSS after endoscopic carpal tunnel release and baseline BCTQ-SSS score. Black circles represent values of individual studies where the circle size is proportional to the study weight in the random-effects model. The red line represents the regression line of best fit with the shaded area indicating the 95% confidence interval. The regression equation to predict BCTQ-SSS change = [0.49 – (0.74 * Baseline BCTQ-SSS)]; p=0.005. BCTQ-SSS, Boston Carpal Tunnel Questionnaire Symptom Severity Scale.

**Supplement Figure 7.** Bubble plot of the association between the change in BCTQ-FSS after endoscopic carpal tunnel release and baseline BCTQ-FSS score. Black circles represent values of individual studies where the circle size is proportional to the study weight in the random-effects model. The red line represents the regression line of best fit with the shaded area indicating the 95% confidence interval. The regression equation to predict BCTQ-FSS change = [0.37 – (0.64 * Baseline BCTQ-FSS)]; p=0.004. BCTQ-FSS, Boston Carpal Tunnel Questionnaire Functional Status Scale.
